# Supplementary material for: Characterization of Nutrient Intake in Biopsy-Confirmed NAFLD Patients
Source: Nutrients. 2022 Aug 23;14(17):3453. doi: 10.3390/nu14173453 (PMC9460347; doi:10.3390/nu14173453)
Supplement: Supplementary file 1 [file nutrients-14-03453-s001.zip › nutrients-1864521-supplementary.pdf]

Supplementary files

Figure S1

The food ingredients list frequency survey sheet used in this study (original seize).

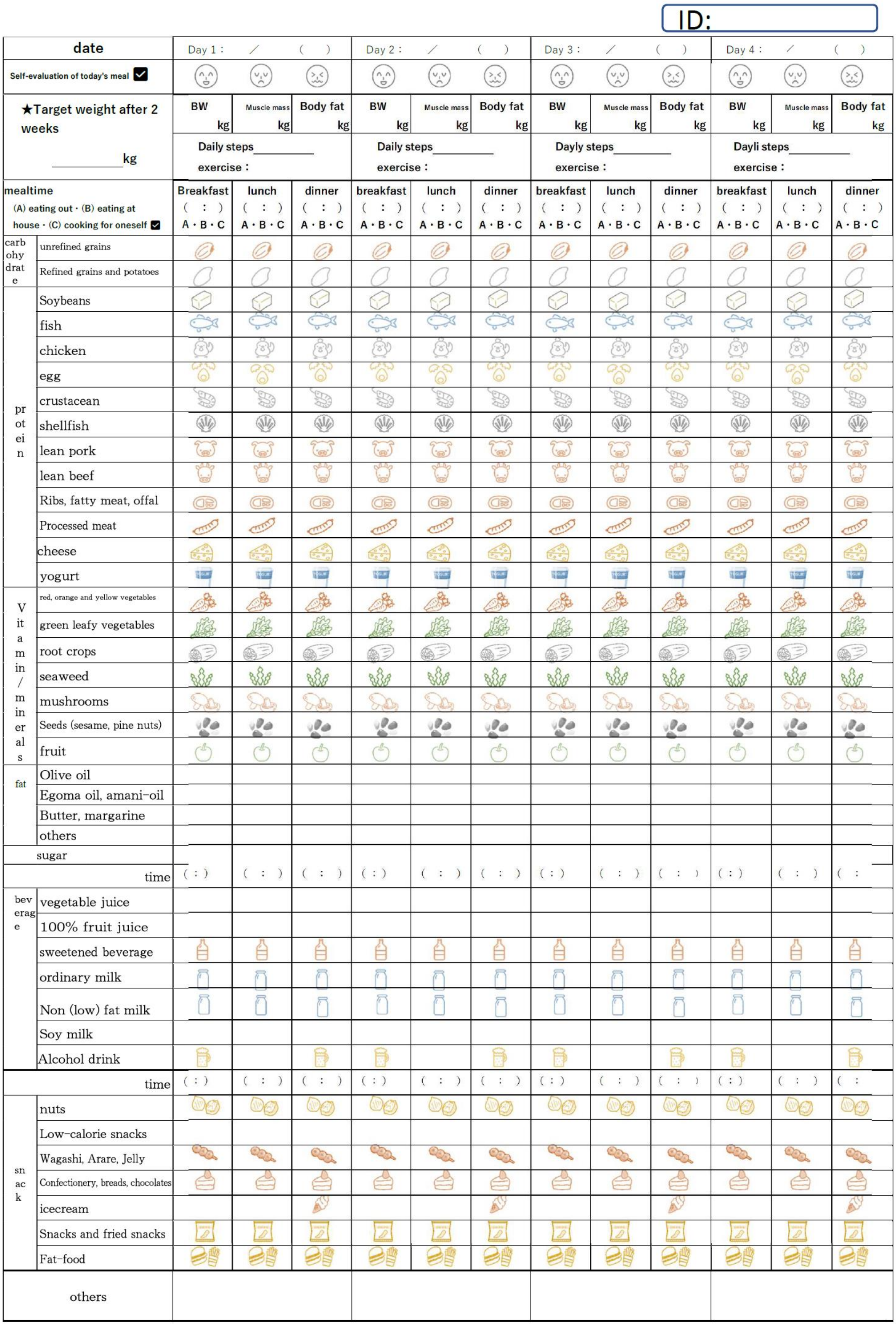

ID:

date		Day 1 : ／ ( )			Day 2 : ／ ( )			Day 3 : ／ ( )			Day 4 : ／ ( )		
Self-evaluation of today's meal ☑													
★Target weight after 2 weeks ____kg		BW kg	Muscle mass kg	Body fat kg	BW kg	Muscle mass kg	Body fat kg	BW kg	Muscle mass kg	Body fat kg	BW kg	Muscle mass kg	Body fat kg
		Daily steps____ exercise :			Daily steps____ exercise :			Dayly steps____ exercise :			Dayli steps____ exercise :		
mealtime (A) eating out・(B) eating at house・(C) cooking for oneself ☑		Breakfast (:) A・B・C	lunch (:) A・B・C	dinner (:) A・B・C	breakfast (:) A・B・C	lunch (:) A・B・C	dinner (:) A・B・C	breakfast (:) A・B・C	lunch (:) A・B・C	dinner (:) A・B・C	breakfast (:) A・B・C	lunch (:) A・B・C	dinner (:) A・B・C
carbohydrate	unrefined grains												
	Refined grains and potatoes												
protein	Soybeans												
	fish												
	chicken												
	egg												
	crustacean												
	shellfish												
	lean pork												
	lean beef												
	Ribs, fatty meat, offal												
	Processed meat												
	cheese												
	yogurt												
Vitamin/minerals	red, orange and yellow vegetables												
	green leafy vegetables												
	root crops												
	seaweed												
	mushrooms												
	Seeds (sesame, pine nuts)												
	fruit												
fat	Olive oil												
	Egoma oil, amani-oil												
	Butter, margarine												
	others												
sugar													
	time	(:)	(:)	(:)	(:)	(:)	(:)	(:)	(:)	(:)	(:)	(:)	(:
beverage	vegetable juice												
	100% fruit juice												
	sweetened beverage												
	ordinary milk												
	Non (low) fat milk												
	Soy milk												
	Alcohol drink												
	time	(:)	(:)	(:)	(:)	(:)	(:)	(:)	(:)	(:)	(:)	(:)	(:
snack	nuts												
	Low-calorie snacks												
	Wagashi, Arare, Jelly												
	Confectionery, breads, chocolates												
	icecream												
	Snacks and fried snacks												
	Fat-food												
others													

Table S1. (a) Comparison of clinical data (male NAFLD group, male control group).

Parameter	Control (n=17)	NAFLD (n=17)	*P* value
**Age (y.o.)**	52.2 ± 10.6	52.2 ± 10.6	n.s.
**BMI**	23.9 ± 3.6	32.4 ± 6.70	0.0001
**SBP (mmHg)**	132 ± 17.7	130 ± 12.6	n.s.
**RBC (×10^4/mm3)**	467 ± 51.7	506 ± 64.2	0.0246
**Plt (×10^4/mm3)**	23.1 ± 5.6	20.7 ± 8.91	0.2755
**TP (g/dL)**	7.3 ± 0.4	7.32 ± 0.452	n.s.
**Alb (g/dL)**	4.4 ± 0.3	4.34 ± 0.294	n.s.
**HbA1c (%)**	5.8 ± 0.7	6.81 ± 0.933	0.0004
**T-Cho (mg/dL)**	200 ± 37.0	188 ± 43.6	n.s.
**TG (mg/dL)**	159 ± 110	169 ± 66.7	n.s.
**AST (U/L)**	26.4 ± 10.9	54.5 ± 31.6	0.0021
**ALT (U/L)**	26.3 ± 17.6	70.8 ± 40.8	0.0004
**GGT (U/L)**	47.7 ± 53.3	114 ± 82.0	0.0055
**Cre (mg/dL)**	0.9 ± 0.5	0.938 ± 0.183	n.s.
**Fe (μg/dL)**	93.8 ± 34.9	125 ± 49.4	0.0231

(b) Comparison of clinical data (female NAFLD group, female control group)

Parameter	Control (n=20)	NAFLD (n=20)	*P* value
**Age (y.o.)**	57.8 ± 10.6	57.8 ± 10.6	n.s.
**BMI**	22.5 ± 3.7	31.0 ± 5.8	0
**SBP (mmHg)**	127 ± 19.1	129 ± 13.2	n.s.
**RBC (×10^4/mm3)**	433 ± 39.3	458 ± 30.3	0.002
**Plt (×10^4/mm3)**	25.1 ± 6.1	21.0 ± 8.23	0.0388
**TP (g/dL)**	7.3 ± 0.4	7.36 ± 0.477	n.s.
**Alb (g/dL)**	4.3 ± 0.3	4.29 ± 0.34	n.s.
**HbA1c (%)**	5.7 ± 0.6	6.68 ± 0.91	0.0004
**T-Cho (mg/dL)**	214 ± 36.0	190 ± 35.5	0.002
**TG (mg/dL)**	132 ± 94.8	188 ± 130	n.s.
**AST (U/L)**	23.0 ± 9.5	51.4 ± 29.1	0.0005
**ALT (U/L)**	19.2 ± 12.2	61.9 ± 38.6	0.0
**GGT (U/L)**	26.3 ± 34.2	112 ± 99.9	0.0037
**Cre (mg/dL)**	0.7 ± 0.3	0.77 ± 0.23	n.s.

**Fe (μg/dL)**	74.2 ± 30.3	113 ± 40.4	0.0007

Table S2. (a) Comparison of nutrient intake data (male NAFLD group, male control group).

Parameter	Control (male)	NAFLD (male)	*P* value
**Total calorie (kcal)**	2141 ± 588.7	2191 ± 464.8	n.s.
**Carbohydrate (g/day)**	275 ± 82.9	277 ± 66.6	n.s.
**Fat (g/day)**	66.3 ± 28.9	80.4 ± 19.6	0.0095
**Protein (g/day)**	78.8 ± 26.2	83.1 ± 18.9	n.s.
**SFA (g/day)**	19.1 ± 9.72	19.2 ± 7.56	n.s.
**mono USFA (g/day)**	24.7 ± 12.1	27.4 ± 8.33	n.s.
**poly USFA (g/day)**	14.3	15.9 ± 49.4	Incalculable*
**Cholesterol (mg/day)**	366 ± 209	360 ± 97.6	n.s.
**Na (mg/day)**	4309 ± 1625	4419 ± 1577	n.s.
**K (mg/day)**	2439 ± 953.9	2196 ± 609.2	n.s.
**Ca (mg/day)**	503 ± 271	460 ± 200	n.s.
**Mg (mg/day)**	270 ± 102	251 ± 78.3	n.s.
**Phosphorus (mg/day)**	1084 ± 372.2	1026 ± 238.9	n.s.
**Fe (mg/day)**	8.30 ± 3.30	9.11 ± 4.24	n.s.
**Zn (mg/day)**	9.20 ± 3.47	8.52 ± 1.93	n.s.
**Cu (mg/day)**	1.23 ± 0.44	1.11 ± 0.251	n.s.
**Vitamin A (μgRAE/day)**	564 ± 1297	416 ± 130	0.0008
**Vitamin B1 (mg/day)**	1.03 ± 0.500	1.19 ± 0.305	n.s.
**Vitamin B2 (mg/day)**	1.25 ± 0.55	1.33 ± 0.677	n.s.
**Vitamin B6 (mg/day)**	1.30 ± 0.55	1.34 ± 0.46	n.s.
**Vitamin B12 (μg/day)**	7.25 ± 8.70	7.25 ± 3.36	n.s.
**Vitamin C (mg/day)**	95.9 ± 71.5	79.5 ± 52.7	n.s.
**Vitamin D (μg/day)**	7.85 ± 9.93	7.05 ± 3.40	n.s.
**Vitamin E (mg/day)**	7.18 ± 3.52	8.69 ± 3.11	n.s.
**Vitamin K2 (μg/day)**	258 ± 192	224 ± 144	n.s.
**Niacin (mgNE/day)**	34.5 ± 13.1	29.2 ± 8.67	0.023
**Folate acid (μg/day)**	310 ± 179	270 ± 113	n.s.
**Pantothenic acid (mg/day)**	6.05 ± 2.27	5.72 ± 1.55	n.s.
**Water-soluble dietary fiber (g/day)**	3.70 ± 1.80	4.65 ± 2.28	n.s.
**Insoluble dietary fiber (g/day)**	12.2 ± 5.17	10.3 ± 3.50	0.0398

**Total dietary fiber (g/day)**	19.9 ± 7.36	19.7 ± 5.45	n.s.
**Salt (g/day)**	10.9 ± 4.13	11.5 ± 3.86	n.s.

* *P* value was not calculable due to impaired data of control subjects

(b) Comparison of nutrient intake data (female NAFLD group, female control group)

Parameter	Control (female)	NAFLD (female)	*P* value
**Total calorie (kcal)**	1717 ± 496.1	1587 ± 352.2	n.s.
**Carbohydrate (g/day)**	226 ± 69.7	203 ± 51.2	n.s.
**Fat (g/day)**	56.7 ± 25.7	56.5 ± 14.6	n.s.
**Protein (g/day)**	66.4 ± 22.4	61.6 ± 14.7	n.s.
**SFA (g/day)**	16.7 ± 8.86	14.4 ± 5.25	n.s.
**mono USFA (g/day)**	20.6 ± 10.4	18.7 ± 6.42	n.s.
**poly USFA (g/day)**	12.1	11.2 ± 4.57	Incalculable*
**Cholesterol (mg/day)**	317 ± 184	327 ± 131	n.s.
**Na (mg/day)**	3651 ± 1411	2981 ± 1076	0.0122
**K (mg/day)**	2273 ± 941.1	2070 ± 660.1	n.s.
**Ca (mg/day)**	494 ± 264	449 ± 133	n.s.
**Mg (mg/day)**	242 ± 94.8	220 ± 62.8	n.s.
**Phosphorus (mg/day)**	948 ± 339	884 ± 232	n.s.
**Fe (mg/day)**	7.53 ± 3.15	7.12 ± 2.10	n.s.
**Zn (mg/day)**	7.67 ± 2.97	6.54 ± 1.68	0.0079
**Cu (mg/day)**	1.07 ± 0.41	0.917 ± 0.261	0.0174
**Vitamin A (μgRAE/day)**	532 ± 906	448 ± 206	n.s.
**Vitamin B1 (mg/day)**	0.88 ± 0.42	0.918 ± 0.446	n.s.
**Vitamin B2 (mg/day)**	1.13 ± 0.50	0.991 ± 0.250	0.0234
**Vitamin B6 (mg/day)**	1.12 ± 0.49	1.14 ± 0.443	n.s.
**Vitamin B12 (μg/day)**	5.90 ± 7.01	5.51 ± 3.47	n.s.
**Vitamin C (mg/day)**	101 ± 75.0	91.7 ± 43.2	n.s.
**Vitamin D (μg/day)**	6.60 ± 7.91	6.27 ± 4.44	n.s.
**Vitamin E (mg/day)**	6.64 ± 3.38	7.46 ± 2.36	n.s.
**Vitamin K2 (μg/day)**	243 ± 185	169 ± 82.1	0.0007
**Niacin (mgNE/day)**	28.6 ± 10.9	22.9 ± 6.61	0.0013
**Folate acid (μg/day)**	295 ± 160	264 ± 101	n.s.
**Pantothenic acid (mg/day)**	5.30 ± 2.05	6.00 ± 4.36	n.s.
**Water-soluble dietary fiber (g/day)**	3.58 ± 1.76	4.01 ± 1.32	n.s.

**Insoluble dietary fiber (g/day)**	11.5 ± 5.24	9.04 ± 2.95	0.0015
**Total dietary fiber (g/day)**	18.0 ± 7.21	14.4 ± 5.03	0.0057
**Salt (g/day)**	9.27 ± 3.58	7.73 ± 2.65	0.0162

* *P* value was not calculable due to impaired data of control subjects
